# Anæsthesia

**Published:** 1885-09

**Authors:** M. G. Jenison

**Affiliations:** Minneapolis


### Anæsthesia.

BY M. G. JENISON, M.D., D.D.S., MINNEAPOLIS.

Read before the Minnesota Dental Society, Aug. 1st.

In this subject of Anæsthesia, we have a condition that is of more or less interest to the whole human race. For, while we exist we must necessarily have a delicate and sensitive nervous system, by which the injury, or abnormal condition of any part is at once conveyed to the brain as a pain.

All through the centuries of time man, has been trying to find some way to escape the penalties of broken laws, and to avoid the pain naturally consequent upon disordered functions, or lesion produced by accident; but until the nature of so called anæsthetics was known his efforts were at the best incomplete and unsatisfactory. It is not merely the endeavor to avoid pain by anæsthetics; but they are given to diminish nervous and mental tax and this means a good deal more than simply avoiding bodily pain. The degree of harm, the apprehension of an operation may vary greatly with different temperaments; in many cases it is worse than the pain itself; the very fear of ill exceeds the ill we fear. Thus we must be governed by the peculiarities of our patients, and as far as possible act accordingly; an anæsthetic, may be required more by one person to have a tooth out, than by another, to have a leg amputated.

The insensibility to pain during an operation diminishes the nervous shock, for persons can and have died of pain; with an advancing civilization the nervous system becomes more sensitive, so the use of anæsthetics is more necessary with the people of the nineteenth century, than with those of past ages.

There are numerous conditions that may be produced, in which a person is more or less insensible to pain, such as coma, syncope, anæsthesia, and sleep. The first three are pathological, the last is the normal condition of recuperation of wasted nerve forces, all these phases, or conditions, are dependent on the nerve supply, and are supposed to be induced by normal or abnormal nerve currents.

There are certain drugs whose office seems wholly connected with the nervous system, known as nerve sedatives, and a distinct class known as anæsthetics.

The history of anæsthetics really dates from the time when a dentist, at his own desire, had a tooth removed without pain, and in the advancement of this part of surgery, the dental profession has always stood in the front rank.

The attempts of the ancients to relieve pain incident to surgical operations are of interest and worthy of a brief review.

Long prior to English civilization, medicine had made considerable progress in Egypt. The physician of that early day, used opium or Indian hemp to render the patient insensible.

In the third century, Hoa Tho, an oriental surgeon, used Indian hemp and hasheesh.

Dioscorides, a Greek writer, mentions the use of Atropia Mandragora a decoction made by boiling mandragora in wine, and of this the patient drank. In 1298 a compound was used of opium cicuta, mandragora, like lactuca and hyoscyamus, to be inhaled from a sponge.

In the school of Laterna, in the south of Italy where medical degrees were first granted to students, Montague, a surgeon, gave a somnific portion to those he proposed to operate on.

A little later on than this, Bartholina says his preceptor used snow, to produce local anæsthesia.

In the last part of the 18th century Lassard, of Paris, gave narcotics to lessen the pains of operation. Early in this century experiments were commenced with oxygen, nitrogen, and their compounds by Priestly, Sir Humphrey Davy, and others.

In 1842-3 Dr. Long, of Athens, Ga. used ether as an anæsthetic, in surgery, but failing to publish his experiments lost much of the credit. In 1844 Dr. Wells, of Hartford, Conn., first used gas for extracting teeth. In 1846 Morton and Jackson, of Boston, profiting by the failures of Wells and others, appear on the list and receive much credit not due them.

Of chloroform, Dr. Guthrie published an account in 1832, stating its therapeutical effects, and the method of obtaining it. But to Prof. Simpson, of Edinburgh, belongs the honor of first demonstrating its effects by inhalation.

During the war of the Rebellion 23,260 persons were operated on under the influence of an anæsthetic, and nearly two-thirds of the cases were under chloroform.

Anæsthetic agents, like other influences that act upon the living, sentient organisms, must be brought in contact with the vital presence to effect their results; and therefore quantity and quality of their application must modify the results desired and attained.

Condition of mind and body should always be taken into consideration, as a normal state of both, conduces to a much more favorable result.

It is seldom satisfactory with a full stomach, nor should it be overlooked, that long wakefulness from pain, or anxiety, complicates and renders uncertain the advanced stages of anæsthesia. The mystery of nerve force and supply, like that of the electric current, which it closely resembles, has never yet been bared to human knowledge.

Experiments have been performed and certain laws deduced, but to these there are so many exceptions that at present it is impossible to say positively, how certain of the principles effect their purpose.

Anæsthesia may be considered as a state of paralyzation of the sensory nerves. It is a complete loss of sensation, and may be produced by various agents. In warm blooded animals by lowering the bodily temperature, in cold blooded by raising it.

The narcotic effect may also be produced by carbonic acid gas, carbonic oxide, coal gas, aldehyde, etc.

The principal, and about the only agents used to produce anæsthesia are nitrous oxide, ether and chloroform. The bromide, and chloride of ethyl, being used to a limited extent.

In mixed anæsthetics, there is little to gain and much to lose. You have nearly the full danger of either separate, and may be rendered careless by supposed safety. That of ether and chloroform is not apt to form a perfect mixture, so that they are administered separately, while the combination of effects is apt to prove confusing, and less likely to be observed and promptly treated.

Vitalized air I object to, on the same grounds, although the amount of chloroform vapor is small.

Ohio Journal, 1883.—Two members suspended from Ohio State Dental Society for advertising " Vitalized Air."

Vitalized air is nothing new, it is a mixture of chloroform and gas in indefinite proportions, was tried nearly twenty years ago, and condemned, as too dangerous for general use. Its recent renewal is due to a charlatan, and those who assist in its use and recommend it as either new, or superior to nitrous oxide, are either themselves deceived or willfully attempting to deceive the public.

How do these agents produce their characteristic effects? Though undisputed demonstrations of their manner of action can be given, there are certain phenomena connected with them that are not so clearly understood.

Claud Bernard says, an anæsthetic is a drug that produces a direct effect upon nerve tissue. Prof. Anistie, that effects are produced by modified blood supply, or alteration of nutrition.

The characteristic effects of common anæsthesia are best produced by inhalation, as they are thereby more readily introduced into the blood. The immense surface presented in the lungs, bringing the blood in osmotic contact with so much of the vapor that the pulmonary circulation becomes completely charged with it, and this is carried to the heart and distributed to every part of the system, without any opportunity being given for its elimination. While if the anæsthetic is injected into a vein, comparatively little effect is produced, as it is carried directly to the heart and to the lungs never reaching the arterioles.

Now comes the question, is it simply held in solution in the serum of the blood, or is it conveyed by the corpuscles. If we answer, in the serum of the blood, that solves the question at once.

But microscopic and other examinations by good authority show that anæsthetics change to a certain extent, the character of the corpuscles; observe the effect of carbonic acid on arterial blood, which it changes to a dark color, while carbonic oxide, has the opposite effect, changing venous blood to a bright red.

Ether mixed with blood, gives a dark purple color, by changing the character of the corpuscles and freeing some of the hematine.

Chloroform turns the blood a brilliant scarlet, thus it may be seen that anæsthetics have power to modify the ability of the blood corpuscles to receive oxygen. Is it in this manner these agents produce their physiological effects? There are other conditions in which the existing phenomena resembles anæsthesia. During sleep for example, as has been proven by experiments on lower animals, we have, first, hyperæmia of the brain, followed by anæmia.

Asphyxia presents many phases similar to anæsthesia. It is a result of deprivation of oxygen, and its effects are mainly manifest upon the medulla.

The lungs have nothing to do with the desire of a suffocating animal for breath; the effect is produced on the nerves by the impure blood being conveyed to the medulla, and hence the desire for more oxygen. As long as this nerve centre is supplied with oxygenated blood, there is no struggle for breath. Though in some respects, asphyxia and anæsthesia resemble each other, there is a wide difference in their pathology, for while one is but the deprivation of oxygen with its characteristic effects, the other has added the positive presence of a toxic agent, which induces an added train of symptoms.

The one is the cessation of the function of the anatomical elements; and the other is partial suspension in some of them.

The one is the complete absence of oxygen from the medulla, the other the partial oxygenation of the tissue elements; here we have two somewhat similar conditions, yet the one is entirely inconsistent with functional activity, and the other quite compatible with life, although it may be prolonged for some time. It does not seem reasonable, that the profound effects of anæsthesia are produced by a change in the form of the blood.

It cannot well be a lack of oxygen, for that is simple asphyxia. We must then conclude that the presence of the anæsthetic agent as carried by the blood does produce a strong impression directly on the nerve tissue, rendering it incompetent to perform its proper function, this would seem to be the case, when we take into consideration the manner of progression, which is from the central ganglion of nerves. Another reason why it would seem that anæsthesia is not produced through a change of nutrition is that its agents effect nervous force to a certain extent, when ordinary function and blood circulation has ceased.

While all of the common anæsthetics produce the same general results, in regard to the sensory nerves, the reflex effect upon the motor system varies widely.

Chloroform and ether produce, first excitement, then complete muscular relaxation. Nitrous oxide generally produces contraction, especially of the muscles of mastication; while most of the other agents produce spasms more or less marked.

The relative safety of anæsthetics has been much discussed, but there seems to be no good reason why any one of them should be excluded in *all* cases.

From well authenticated records, the proportion of fatal cases is about as follows:

| | | |
|---|---|---|
| Chloroform ........ | 1 in | 2,872. |
| Ether................ | 1 in | 23,203. |
| Nitrous Oxide...... | 1 in | 100,000. |

That we cannot with impunity so far interfere with a normal existence, as to suspend some of the most important functions of life, must be apparent to all. The progress of anæsthesia is gradual, it does not overwhelm the whole nervous system at once, but conquers in detail, this seems to be the order of its attack upon the great nervous centres:

1. The Central Hemispheres.
2. Spinal Cord.
3. Ganglionic system.
4. The Respiratory ganglia.

Certain definite quantities of any toxic agent, may be tolerated by the human economy, but that point once reached, and passed, the peril commences. Were this point a definitely fixed one, we might know when it was approached, and avoid exceeding it, but the line of safety depends greatly upon modifying circumstances.

An intemperate person incurs a greater risk than one who is temperate, although alcohol is often given to one. that anæsthesia may be more easily established. A person who is readily influenced by an anæsthetic is more liable to death than one who is not.

In producing anæsthesia we desire to produce narcosis of the whole nervous system with the exception of that presiding over involuntary motion; the danger consists in going too far, and overcoming this also, so that common prudence bids us proceed with great caution. On the other hand if the narcosis is not carried far enough, sufficient shock may be produced by surgical operations to destroy the life of the patient.

The great danger in the administration of any anæsthetic (nitrous oxide excepted) lies in allowing the patient to breathe an atmosphere too highly charged with it. Experiments have proved, that not more than five per cent. of chloroform and ether can be breathed without danger; and observations of Sallem and Perrin and Duray have demonstrated that an atmosphere of eight per cent. of chloroform is fatal, while two per cent. can be breathed a long time without producing anæsthesia.

It is better to use these agents with an inhaler which can be implicitly relied upon to properly dilute the vapor.

In the use of nitrous oxide, safety lies in withdrawing it before narcosis shall have proceeded to far.

The first stage of anæsthesia is hyperæsthesia, though varying in intensity. There is an increased flow of blood, through an accelerated action of the heart, and dilation of the capillaries with contraction of the iris.

This is especially marked in exhibitions of chloroform, and during its administration, as well as that of ether, the excitement is often very violent; it is at this time that danger is imminent in cases of lesion of the heart. Under the stimulus, if the muscular tissue of the heart is weakened by fatty degeneration, or if there be marked diminution of its left ventricular capacity, its spasmodic struggles may result in an embolism; when as is usually the case, the muscular coats of the arteries are weakened through an irregular action of the heart, or that organ itself may become paralized, through its extraordinary effort to respond to the exertion demanded; death then occurs through spasm of the heart, or paralysis of the sympathetic nerve.

The most reliable statistics show that more deaths occur at this stage of chloroformism than at any other, and death has generally resulted from pushing the effects beyond the bounds of prudence, or in allowing an atmosphere too highly charged with the vapor.

If chloroform is properly administered and closely watched, it is not as dangerous as has been represented; but as it is more powerful than the other agents, it requires greater care and skill in its administration.

The excitement stage of ether narcosis, is attended with much less danger than that of chloroform. Death from ether, generally occurring from paralysis of the muscles of respiration, or the filling of the smaller bronchi with mucus, through the destruction of the sensibility of the membrane, and the consequent cessation of the ciliary motion of the villi.

In nearly all cases of death from ether, it will be found, that bronchial si gns, andstertorous breathing, precede death. Nitrous oxide is extensively used by dentists, and for some of the minor operations in general surgery, and is considered the safest of all anæsthetics.

In its use, the atmosphere is entirely excluded and this takes its place in the blood, though it is thought not to change its combination of $N_2O$. It acts rapidly and its effects soon pass off, the average time being from 50 to 100 seconds.

The signs of danger are:

Hurried respiration,
Labored "
Gasping "
Stertorous "
Suspension of "

Weak pulse.
Irregular "
Intermittent "
Fluttering "
Pulselessness.
Extreme dilation of pupils.
Darkened appearance of blood from incision.
Cessation of hemorrhage.
Falling of lower jaw.
Vomiting.
Relaxation of sphincters.
Lividity of face.
Great pallor of face or lips.

The progress of anæsthesia, according to Anistie, is in its effects, manifested :

1. Upon cerebral hemispheres.
2. Cerebellum.
3. Medulla oblongata.

The patient will lose :

1. Local sensibility in the extremities.
2. Intellectual powers.
3. General power.
4. Power of receiving sensory impressions.
5. Power of respiration.
6. Involuntary muscular action.

There seems to be, also, a general progress of anæsthesia, through the cerebral nerves, from the anterior portion of the brain posteriorly,

The olfactory being the first to succumb, as is shown by the demand for more of the agent. This is easily accounted for by the fact, that the anæsthetic reaches the terminal filaments of this nerve before it is presented to any of the others.

Then follow loss of sight, etc.

The 10, 11, 12, nerves being the last to succumb.

In cases of danger from ether, resort principally to artificial respiration. With chloroform use every effort to stimulate the heart; in both cases keep up the temperature.

Many a life has been lost by allowing the temperature to be reduced by the subsidence of the functional activity. Several cases are on record where by proper and persistent treatment, patients have been brought back to life, long after they have been pronounced irrecoverably dead. With nitrous oxide, the principal thing, is to admit plenty of fresh air, and resort to artificial respiration, in some few cases, electricity and other stimulants may be demanded. In all cases, put the patient in a horizonital position. In administering any anæsthetic, there are certain precautions that should always be observed. First have the room as quiet as possible and the absence of everything tending to annoy or excite the patient, as far as possible keep their mind from the operation, see that proper restoratives are within reach, and never operate without an assistant. See that clothing is loose and respiration easy, that the stomach is not loaded, and that no foreign substance is in the mouth.

Note as far as possible any peculiarities or abnormal conditions.

The administration of an anæsthetic should be intrusted to a competent assistant. He has his duty to perform, while the operator does *his* work ; one pair of eyes, one pair of hands, and one brain, is not sufficient, safe, or justifiable.

Many can never administer anæsthetics satisfactorily, requiring as it does, composure, determination, and a confidence resulting from a thorough knowledge of the proceeding.

To such a man, patients readily yield, where they would fear and oppose a nervous, undecided operator. Never was a greater boon discovered than anæsthesia. I would not advocate its indiscriminate use. All surgical operations should be rendered as free from pain as possible. So with a clear understanding of what we are doing, we can relieve our patients of pain which they dread, make severe operations easy, and still not advertise ourselves as "painless" frauds and public butchers.

